# Biological activity of silver nanoparticles synthesized from untapped secondary metabolites of ***Olea europea*** endophytic ***Bacillus amyloliquefaciens***

**DOI:** 10.1371/journal.pone.0321134

**Published:** 2025-05-07

**Authors:** Fawad Hayat, Sumera Afzal Khan, Muddasir Khan, Zahoor Khan, Ho-Youn Kim, Abdul Qadeer, Khalid J. Alzahrani, Fuad M. Alzahrani, Muhammad Hamayun

**Affiliations:** 1 Center of Biotechnology and Microbiology, University of Peshawar, Peshawar, Pakistan; 2 Formulatrix Pakistan (Pvt.) Ltd., Lahore, Pakistan; 3 Convergence Research Center for Smart Farm Solution, Korea Institute of Science and Technology (KIST), Seoul, Korea; 4 Department of Cell Biology, School of Life Sciences, Central South University, Changsha, China; 5 Department of Clinical Laboratories Sciences, College of Applied Medical Sciences, Taif University, Taif, Saudi Arabia; 6 Department of Botany, Abdul Wali Khan University, Mardan, Pakistan; Al-Azhar University, EGYPT

## Abstract

Endophytic microbes offer frequent contributions to the identification of novel biologically active compounds. The current study aimed to isolate untapped *Olea europaea-associated Bacillus amyloliquefaciens* and to explore their bioactive secondary metabolites as well as its mediated silver nanoparticles (AgNPs). *B. amyloliquefaciens* analysis revealed the existence of 23 compounds in metabolite extract by using Gas chromatography-mass spectrometry (GC-MS) analysis*.* AgNPs were synthesized by using metabolite extract and confirmed by using UV-Vis spectrophotometry, Energy Dispersive Spectroscopy (EDS), and Fourier Transform Infrared Spectroscopy (FTIR). *B. amyloliquefaciens* metabolite extract displays a high inhibition zone (19 mm) against *Morganella morganii* and *Escherichia coli*, while AgNPs exhibit high inhibition zone (22 mm) against *M. morganii*. The extract shows 64.8% and AgNPs display 99.8% antioxidant activity in 5mg/mL concentration. In analgesic effect, after 90 minutes at 100 mg/mL concentration, the extract shows a mean latency time of 17 seconds while AgNPs show 19 seconds, respectively. In conclusion, *B. amyloliquefaciens* metabolite extract and AgNPs exhibited significant antibacterial, antioxidant, and analgesic activities in various concentrations while comparatively AgNPs displayed higher bioactive potential. Endophytic bacteria associated with *O. europaea* have diverse bioactive metabolites with promising pharmaceutical activities, as well as their mediated AgNPs increase these activities. Further research on the exploration of endophytic bacterial metabolites and its mediated nanoparticles will prompt the discovery of novel bioactive compounds.

## Introduction

Endophytes are microorganisms that live inside the plant tissues without causing damage to the host. They are abundantly present in rainforest plants, and occupy 7% of the earth’s land surface [1]. The International Union for Conservation of Nature and Natural Resources (IUCN) data suggest that there are 297,326 species of plants including monocotyledons (59,300 species), dicotyledons (199,350 species), and gymnosperms (979). However, only a small number of endophytes associated with these plant species have been studied to date [1]. They exist in almost every plant on the earth [2], while their isolation from soil has become more difficult due to their symbiotic associations with other organisms [3]. Research on endophytic microbes has contributed to the discovery of new biologically active molecules [3]. They promote plant growth, health, and beneficial effects. Moreover, these are potentially exploitable for biotechnological processes, such as the development of new probiotics, enzymes, and polymers with pharmaceutical and industrial applications [4–6]. The secondary metabolites produced by endophytes can be implemented as therapeutics in the pharmaceutical and agricultural industries. Natural compounds from endophytes exhibit activities including; antibacterial, antifungal, anticarcinogen, immunosuppressant, and antioxidant [4].

Secondary metabolites are organic chemicals produced by plants, bacteria, or fungi that are not directly involved in the organism’s regular growth, development, and reproduction. It is not required for bacteria to flourish, but it does help them to interact more effectively with their environment. Endophytic bacteria especially *Bacillus* sp. produce terpenoids, alkaloids, steroids, peptides, polyketones, flavonoids, quinols, and phenols that aid plant-bacteria interactions and colonization [7]. These metabolites shows various biological activities like anticancer, antioxidant, antibacterial, anti-inflammatory, and immunosuppressive properties [6]. Moreover, the biologically synthesized silver nanoparticles (AgNPs) have more compatibility and potential than chemically synthesized nanoparticles. The endophyte-mediated nanoparticles were reported in the last few years, opening a new research interface for scientists in medical sciences. However, different areas of research are still in need of exploitation and further studies for their functionalization [8].

Olive is one of the most ancient domestic cultivated plants. They found in two forms namely wild (*Olea europaea* subsp. europaea var. sylvestris) and cultivated (*Olea europaea* L. subsp. europaea var. europaea) [9]. Their leaves were traditionally used in the treatment of diabetes, malaria, hypertension, coughs, asthma, lumbago, rheumatism, kidney problems, urinary tract infections, nose bleeding, eye infection, and to relieve sore throats as well as antioxidant [9–12]. Endophytes associated with Olea europaea have not been previously reported. Due to the high pharmaceutical potential of this plant and the untapped bioactive potential of its associated endophytic bacteria, the current study was conducted. We aimed to isolate *Bacillus amyloliquefaciens* from Olea europaea and investigate the bioactive potential of its secondary metabolites. Additionally, the study aimed to evaluate the potential of AgNPs synthesized using these metabolite extracts.

## Materials and methods

### Plant collection and isolation of endophytic bacteria

**Fig 1:**
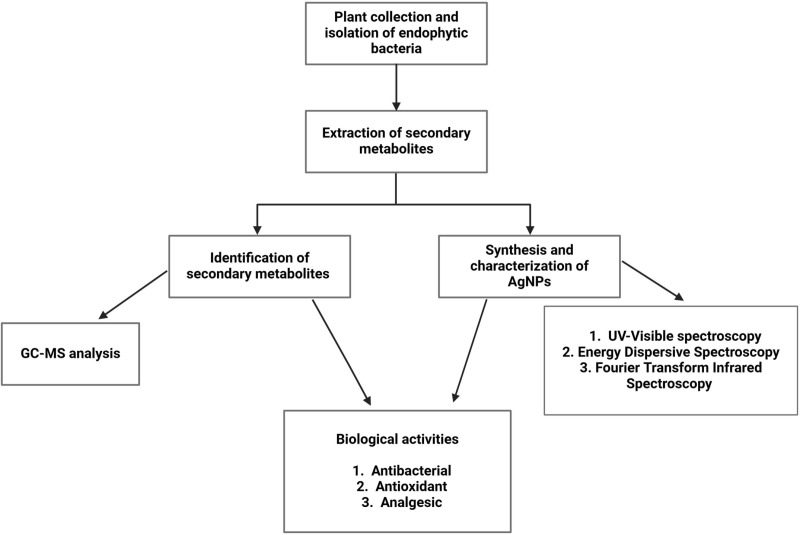
Experimental flow chart of the study.

Healthy *O. europaea* plants were selected from two distinct areas, Waziristan and Jamrud, located in Khyber Pakhtunkhwa, Pakistan. The random samples of its aerial parts, including soft branches, leaves, and fruits were collected in sterilized zipper bags. Samples were transported for processing (Fig 1) to microbiology research laboratory in Center of Biotechnology and Microbiology, University of Peshawar, Pakistan. The samples were first washed under tap water to remove dust or dirt, if any, and then surface sterilized by standard procedure [13]. Briefly, the samples were rinsed with tween 20 for 10 minutes and then washed with distilled water. Then dipped into 70% alcohol for 25–30 seconds and washed again with distilled water. The cleaned samples were further treated with sterile phosphate-buffer saline (PBS) as per protocol and left to air dry in a laminar flow hood. The sterilized parts were sliced into 0.5–1.0 cm pieces with the help of a sterilized blade and placed carefully on nutrient agar plates. The plates were incubated at 37˚C for 24–48 hours. After incubation each bacterial colony that appears close to the plant tissue was carefully picked and streaked on fresh nutrient agar plates [13,14].

### Identification of targeted endophytic bacteria

Morphologically distinct colonies were identified by observing colony characteristics such as Gram stain nature, color, and shape [15]. The target isolate was selected, cultured, purified, and stored in the laboratory at -80˚C in glycerol stock (50%) solution for further studies. For molecular identification, the bacterial genomic DNA was extracted using the DNA purification kit Genejet and stored at -20˚C. The DNA concentration was measured by NanoDrop. The extracted DNA was sent to Macrogen, Korea for *16SrRNA* gene sequencing. The obtained raw sequence was BLAST on NCBI (National Center of Biotechnology Information) and phylogenetic analysis was done on Mega 11 software [14].

### Extraction and identification of secondary metabolites

Bacterial isolate was inoculated in a flask containing 5 L of nutrient broth (NB) and incubated for 4–5 days at 110 rpm on a rotary shaker at 37 ˚C. After fermentation, the culture broth was filtered with Whatsman filter paper. Then, the filtrate was extracted three times with ethyl acetate. The ethyl acetate was evaporated by using a rotary evaporator. The yield of the extract was determined and recorded. The crude extract was analyzed by Gas chromatography-mass spectrometry (GC-MS) to identify the compound present in the extract [14,15].

### Characterization of silver nanoparticles (AgNPs) synthesized from secondary metabolites

For the synthesis of AgNPs from *O. europaea* metabolite extract, the hydrothermal method was used [16]. A stock solution (5 mM) of silver nitrate was prepared using 100 mL sterile distilled water. Then, 1 mL of 5 mM silver nitrate solution was added into 10 mL of extract. The control was prepared without the addition of silver nitrate. The solution was subjected to an autoclave at 121^0^C for 15 minutes. UV-visible spectroscopy was employed to analyze the spectral properties of the silver nanoparticles (AgNPs) across a wavelength range of 200–800 nanometers. Scanning Electron Microscopy (SEM) and Energy Dispersive Spectroscopy (EDS) were also conducted concurrently to determine the topology and elemental composition of the particles. Fourier Transform Infrared Spectroscopy (FTIR) was used to identify the possible biomolecules involved in the reduction and capping of AgNPs. It was first freeze-dried and then finely ground with potassium bromide to form pellets suitable for FTIR analysis. An FTIR spectrum was recorded using a VERTEX 70 Spectrometer (Japan) for insights into the functional groups present on the nanoparticle surfaces. These functional groups play a critical role in the biosynthesis and stabilization of the AgNPs, as they may indicate the presence of various organic molecules acting as capping agents. Furthermore, the zeta potential of the colloidal particles in water was measured using a Zetasizer Ultra (Malvern Panalytical, United Kingdom) [16,17].

### Bioactivities of metabolites extract and synthesized AgNPs

#### Antibacterial.

By using the agar well diffusion method [18], the secondary metabolite extract and synthesized AgNPs in 2 mg/mL concentration were suspended in dimethyl sulfoxide (DMSO) and were screen against some bacterial pathogenic strains; *Morgonella morganii, Klebseilla* sp., *Pseudomonas aeruginosa, Escherichia coli, Enterobacteriaceae*. These strains were obtained from Hayatabad medical complex and Khyber teaching hospital. Amoxicillin antibiotic was used as positive control and DMSO as a negative control.

#### 2,2-diphenyl-1-picrylhydrazyl (DPPH) radical scavenging.

DPPH free radicals are highly stable and widely used to evaluate the radical scavenging activity of the antioxidants. Scavenging activity is based upon the reduction of DPPH radicals by hydrogen-donating antioxidant compounds by forming DPPH-H. This activity was performed followed by the method described by [19] with minor changes. Concentrations (1, 3, and 5 mg/mL) of secondary metabolite extract and synthesized AgNPs were dissolved in methanol and reacted with freshly prepared DPPH solution (60 mM, Sigma). The reaction mixture was incubated for 30 minutes in a dark environment. The absorbance was measured at 520 nm, while decreasing absorbance of the DPPH solution indicates an increase in radical scavenging activity. The scavenging activity (%) was calculated using the following equation;


DPPH scavenging activity (%)=[(Ao/A1)/Ao]×100

Whereas Ao is the absorbance of control (blank) and A1 is the absorbance of the sample. Methanol was used as a blank whereas ascorbic acid (Sigma) was used as the reference compound [18].

#### Analgesic.

This activity was ethically approved vide no. REB-12 and performed at the Department of Pharmacy, University of Peshawar, Pakistan according to internationally accepted standards, followed by the described method [20,21]. Modest changes were made to Eddy’s hot plate test. In this study, male albino mice were employed. Two hours before the start of the test, the mice were taken off from their diet. The mice had a pre-test on a hot plate maintained at 55 ± 1 °C. To reduce discrepancies, the animals were not allowed to have latency times longer than fifteen seconds. There were five animals in each group and six groups total from these species. Group I received intra-peritoneal injections of 10 mL/kg normal saline (NS). Group II received treatment with the reference medication diclofenac sodium (50 mg/kg). The test samples (secondary metabolite extract and synthesized AgNPs) were injected into Groups III, IV, V, and VI at doses of 50 and 100 mg/kg, respectively. Following intra-peritoneal treatment, the amount of time (measured in seconds), until the animal lifted, clicked, leaped, or licked its forepaws, was noted after 30, 60, and 90 minutes. Thirty seconds was the cut-off period to prevent tissue injury.

### Statistical analysis

All bioactivities were performed in triplicate and the means of the obtained results were analyzed by using a One-way ANOVA test to calculate the significance (p<0.05). The GraphPad Prism 10.4.1 software was used to analyze the data.

## Results and discussion

### Isolation and identification of *Bacillus amyloliquefaciens*

**Fig 2:**
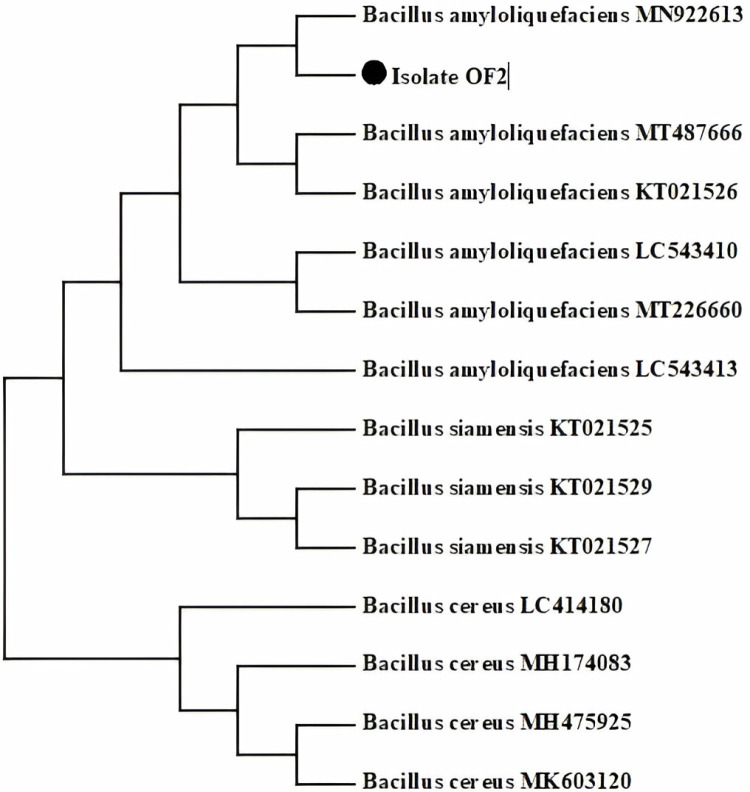
OF2 isolate phylogenetic tree generated by using MEGA 11 software related to *B. amyloliquefaciens.*

Healthy *O. europaea* plant samples (S1 Fig) were washed to remove dust or dirt, if any and then surface sterilized by submerging in 70% ethanol solution (5–10 minutes), followed by rinsing using double deionized distilled water. These were then air-dried in a laminar flow hood, sliced into small pieces (0.5–1.0 cm), and placed on nutrient agar plates (S2 Fig). The plates were then incubated at 37°C for 24 hours. Repeated sub-culturing of bacterial isolates on fresh nutrient agar plates resulted in the purification of bacterial isolates. Preliminarily, among the isolated colonies the targeted *B. amyloliquefaciens* was identified as rod shape, Gram-positive, catalase test positive, spore-forming, and was named as OF2. The pure culture of OF2 was stored on agar slants at 4°C, while the nutrient agar plate’s culture was used for molecular identification. The DNA was extracted using DNA purification kit Genejet and sent to Macrogen, Korea for *16SrRNA* gene sequencing (S3 Fig). The resultant NCBI Nucleotide BLAST analysis of OF2 isolate showed its close evolutionary relation with the *B. amyloliquefaciens* strain (Fig 2). Similarly, according to Singh et al. [22], *Bacillus* species predominate among the endophytic, consistent with our findings. The fact that *Bacillus* sp. are consistently found in many investigations highlights their importance as endophytes and provides opportunities for more investigation into their functional functions and potential applications in biotechnology and agriculture [23,24].

### Production and extraction of secondary metabolites from *B. amyloliquefaciens*

After the identification, the secondary metabolites were extracted using ethyl acetate as the solvent. The bacterial strain produced around 200 mg of the secondary metabolites extract. A comparison of these findings with previous investigations by [13,25] indicates several significant parallels and divergences. In line with accepted practices in microbial ecology research, ethyl acetate is used as a solvent to extract secondary metabolites from endophytic bacteria. The successful completion of isolating pure cultures of endophytic bacteria and extracting secondary metabolites from them is consistent with earlier research [13,14,25] that revealed similar procedures for isolating bioactive compounds from microbial cultures. The isolated endophytic bacteria produced a significant number of secondary metabolites, as evidenced by the yield of about 200 mg.

### Identification of secondary metabolites via GC-MS

Since the *Bacillus* genus has a very active metabolism, it is best recognized for producing some of the most well-known natural compounds. *Bacillus* species also produce cyclic lipopeptide antibiotics, iturin, surfactin, and fengycin, streptavidin, bacillomycin D, and surfactin [26]. In accordance with the GC-MS study of *Bacillus* sp. metabolites by Naveed et al. [27], they found 1,2-benzenedicarboxylic acid, 1,1-butoxy-1-isobutoxy-butane, 2-propanone, 3,3-ethoxycarbonyl-5-hydroxytetrahydropyran-2-one, and 4,4-ethylenedioxy-1-pentylamine. Presently, the GC-MS analysis revealed the existence of 23 compounds in the OF2 isolate secondary metabolites extract. The most dominated compound was propanedioic acid, phenyl- (R.T: 9.606) with a concentration of 22.83%, 2,3-butanediol, monooxime (R.T: 6.224) detected at a concentration of 11.58%, pyrrolo[1,2-a]pyrazine-1,4-dione and hexahydro-3-(2-methyl) (R.T: 22.633) found at concentration of 12.61%, 1-hexanol, 2-ethyl- (R.T: 4.697) present at a concentration of 6.53%, 2-methyl-4-phenyl-1-pyrrolidin-1-yl-butanne-1,4-dione (R.T: 20.283) detected at a concentration of 8.43%, and 4-bromobutanoic acid, phenyl ester (R.T: 23.436) present at a concentration of 5.98%. These results indicate the presence of diverse compounds in the OF2 secondary metabolites extract, while propanedioic acid, phenyl- being the most abundant compound (Table 1).

**Table 1:** GC-MS results of the 23 secondary metabolites present in the extract of OF2 isolate, identified using GC-MS QP2010 Plus (Shimadzu, Japan).

S. No.	Compounds Name	Retention Time	Peaks Area	Concentrations (%)
1.	1-Hexanol, 2-ethyl-	4.697	2707740	6.53
2.	2,3-Butanediol, monooxime	6.224	4796513	11.58
3.	Propanedioic acid, phenyl-	9.606	9458417	22.83
4.	Phenol, 2-methyl-5-(1-methylethyl)-	10.376	1432764	3.46
5.	2,4,4-Trimethylbut-2-enolide	10.569	971449	2.34
6.	Octadecane, 6-methyl-	11.800	464872	1.12
8.	3-Hexadecyloxycarbonyl-5-(2-hydroxyethyl)-4-methylim	11.800	387485	0.94
9.	Ketone, vinyl-pyrrolidinyl-	14.278	1126222	2.72
10.	4-(Prop-2-enoyloxy)tridecane	14.575	1588231	3.83
11.	1-Tridecanol	14.808	727300	1.76
12.	Isolongifolene, 4,5,9,10-dehydro-	15.875	191443	0.46
13.	Nonadecanoic acid	16.931	405356	0.98
14.	Hexadecane	17.772	638158	1.54
15.	1-Decanol, 5,9-dimethyl-	17.960	696889	1.68
17.	Tetradecane	20.026	1265594	3.05
18.	2-Methyl-4-phenyl-1-pyrrolidin-1-yl-butanne-1,4-dione	20.283	3492201	8.43
19.	Pyrrolo[1,2-a]pyrazine-1,4-dione, hexahydro-3-(2-methyl)	22.633	5225340	12.61
21.	4-Bromobutanoic acid, phenyl ester	23.436	2479227	5.98
22.	Tetradecanoic acid	25.632	2123682	5.13
23.	6-Octadecenoic acid, methyl ester, (Z)-	28.117	1257096	3.03

### Synthesized AgNPs from secondary metabolites of *B. amyloliquefaciens*

Synthesis of AgNPs was achieved by the appearance of the dark brown color in the reaction solution. This color transformation is a typical feature of the reduction of silver ions to metallic silver nanoparticles. The successful synthesis of AgNPs reflects the bio-reducing potential of secondary metabolites from endophytic bacteria. The use of secondary metabolites as a reducing agent aligns with the green synthesis methodology utilized in numerous researches [28,29]. Green synthesis techniques that employ organic compounds or biological agents have drawn a considerable amount of interest because of their potential uses in biomedicine and nanotechnology as well as their environmental friendliness. One frequent feature linked to the reduction of silver ions to metallic silver nanoparticles is the color change in the reaction solution from light yellow to dark brown upon AgNPs production. This shift is a trustworthy marker for nanoparticle production and acts as a visual key for the formation of AgNPs [29]. The successful synthesis of AgNPs using secondary metabolites from endophytic bacteria highlights the potential of these compounds as effective reducing agents in nanoparticle synthesis. This finding aligns with previous research [8] that has demonstrated the diverse role of secondary metabolites in mediating the biosynthesis of nanoparticles with various applications, including antimicrobial, anticancer, and catalytic properties.

### Characterization of synthesized AgNPs

**Fig 3:**
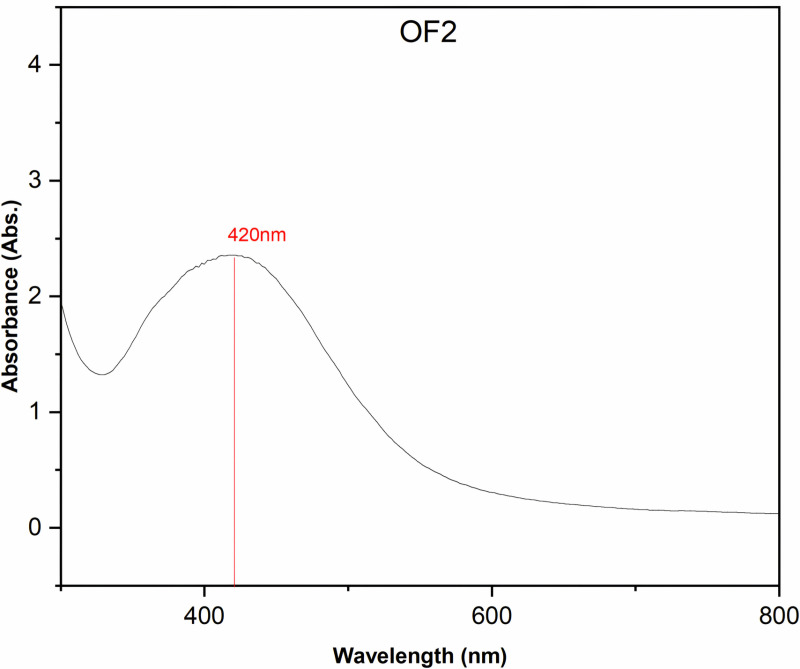
The UV-VIS spectrophotometric analysis of AgNPs, synthesized by using OF2 secondary metabolites extract, revealed distinct Plasmon resonance peaks at 420nm.

The colloidal solution of AgNPs was screened for maximum absorption (200–800 nm) using UV-VIS spectrophotometer. The analysis revealed distinct plasmon resonance peaks at 420 nm (Fig 3). In agreement, a plasmon resonance peak at a wavelength ranging between 400–430 nm, confirming the biosynthesis of nanoparticles [30]. The presence of these peaks is a strong indicator of the successful bio-reduction of silver ions (Ag⁺) to silver nanoparticles (Ag⁰) in the tested sample. This phenomenon occurs because AgNPs exhibit unique optical properties, primarily due to the collective oscillation of their conduction electrons when excited by light, known as surface plasmon resonance (SPR). The observed plasmon resonance peaks for the biosynthesized AgNPs are consistent with findings from previous studies. For instance, similar peaks have been reported for various silver nanoparticles at a wavelength of 426 nm [30], within the range of 400–470 nm [31], at 420 nm [32], and at 410 nm [16]. These results reinforce the reliability of the current biosynthesis methods and suggest that the AgNPs produced in this study possess comparable properties to those reported in earlier research. This alignment with previous literature not only validates the bio-reduction process used but also highlights the reproducibility and stability of the biosynthesized AgNPs.

**Fig 4:**
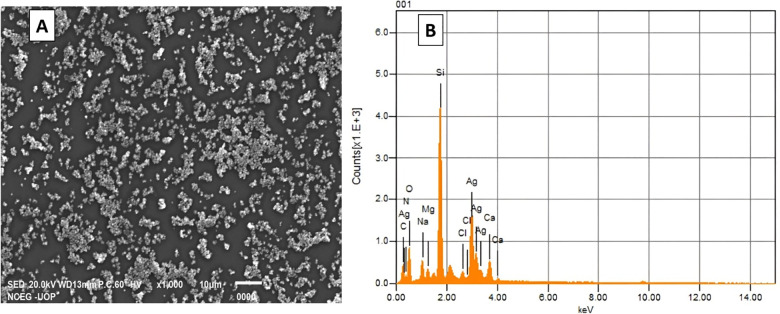
SEM micrograph of AgNPs at 1000x magnification (A) and EDS elemental spectrum of biosynthesized AgNPs (B).

SEM analysis revealed that the AgNPs exhibited a spherical and rough morphology and appeared to cluster into larger aggregates with an average size of 24 nm (Fig 4A). EDS analysis further confirmed the presence of silver in the sample. The EDS spectra displayed characteristic peaks for silver within the energy range of 3.00–3.5 keV, corresponding to approximately 50–350 counts (Fig 4B). These peaks are indicative of the elemental composition of the AgNPs, providing additional confirmation of their successful synthesis. The presence of these peaks aligns with the expected energy values for silver, confirming the formation of AgNPs in the sample. This analysis offers a comprehensive understanding of the shape, size, and elemental composition of the biosynthesized AgNPs, supporting their characterization and potential applications [16,31,33].

**Fig 5:**
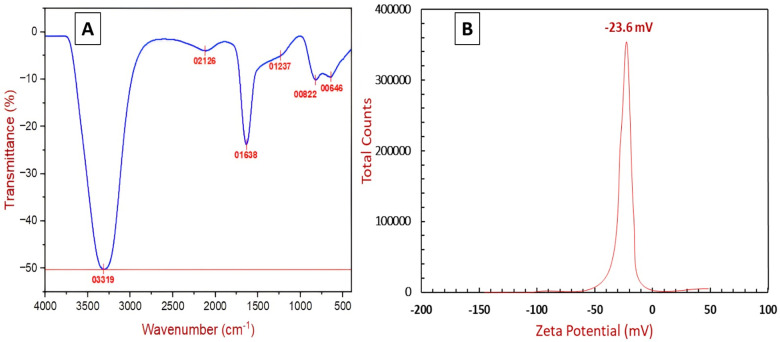
The FTIR spectrum (A) and Zeta potential (B) of AgNPs synthesized by using OF2 secondary metabolites extract.

FTIR analysis of synthesized AgNPs showed an intricate spectrum with six distinct peaks, shown in Fig 5A, at 3319 cm^-1^, 2126 cm^-1^, 1638 cm^-1^, 1237 cm-1, 822 cm^-1^, and 646 cm^-1^. The existence of these peaks indicates that there were different functional groups in the sample. The peak at 3319 cm^-1^ lies in the broad region and is usually occupied by O-H stretching vibrations. This could be a sign of alcohols, phenols, carboxylic acids, or even amines with primary or secondary amines. The peak at 2126 cm^-1^ was an unusual region and could be the functional group of many others. The peak at 1638 cm-1 was clear proof of a carbonyl (C=O) functional group. This group encompassed ketones, aldehydes, amides, and carboxylic acids. The peak at 1237 cm^-1^ might be the result of the C-O stretching vibrations. This may be a sign of the existence of several functional groups that have C-O bonds, for example, alcohols, ethers, or esters. The tops at 822 cm^-1^ and 646 cm^-1^ are usually assigned to the bending vibrations of different functional groups. This FTIR spectrum indicates the presence of a molecule with a carbonyl group (C=O) and other possible functional groups like O-H and C-O. This study agrees with the findings of earlier studies [34,35]. The zeta potential of the synthesized silver nanoparticles (AgNPs) was found to be -23.5 mV (Fig 5B) Zeta potential is a crucial parameter in determining the stability of colloidal dispersions, as it reflects the surface charge and the electrostatic repulsion between particles. A value greater than ±20 mV for these particles indicates good stability due to sufficient repulsive forces preventing particle aggregation [36].

### Bioactivities of metabolites extract and synthesized AgNPs

#### Antibacterial.

The examined bacterial strain exhibited significant (p<0.05) levels of antibacterial activity. Besides this, AgNPs successfully increased the zones for antibacterial activity, as details presented in Table 2. As among the bacterial pathogens, OF2 extract display high inhibition zone (19 mm) against *M. morganii* and *E. coli*, while OF2-AgNPs exhibit high inhibition zone (22 mm) against *M. morganii*. Similarly, previous study also described the antibacterial activity of endophytic bacterial metabolites [14]. Moreover, previously also reported that microbial secondary metabolite mediated AgNPs enhance the antibacterial effect [37]. Furthermore, the most dominant chemical was determined to be propanedioic acid, phenyl-, with a concentration of 22.83%, which is well recognized for its antibacterial action [38].

**Table 2:** Antibacterial activity of OF2 secondary metabolites extract and OF2-AgNPs determined by agar well diffusion method at 2 mg/mL concentration (S1 Table).

S. No.	*Enterobacteriaceae*	*Klebsiella* sp.	*M. morganii*	*E. coli*	*P. aeruginosa*
**Zone of inhibition (mm ± SD)**				
OF2 extract	13 ± 0.354	14 ± 0.707	19 ± 1.061	19 ± 0.707	16 ± 0.707
OF2-AgNPs	14 ± 0.707	16 ± 1.414	22 ± 1.768	20 ± 1.768	17 ± 1.061
Positive control	20 ± 0.354	19 ± 0.354	12 ± 0.707	24 ± 1.414	10 ± 0.354
Negative control	0	0	0	0	0

OF2 extract and OF2-AgNPs demonstrated significant (p<0.05) antibacterial effects.

#### DPPH radical scavenging.

**Fig 6:**
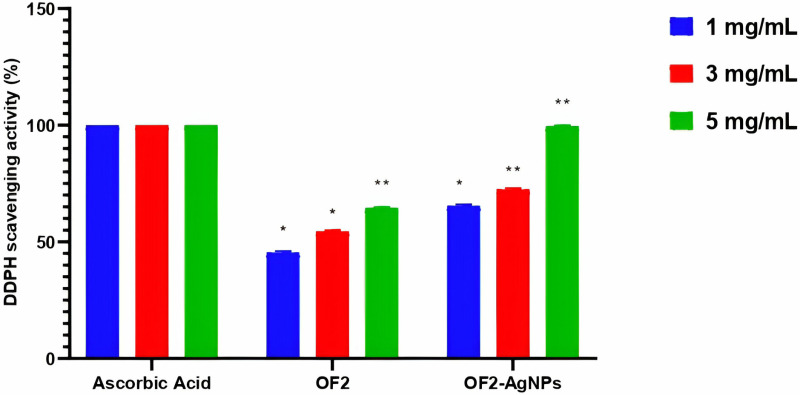
DDPH radicals scavenging activity (%) of *B. amyloliquefaciens* derived secondary metabolites (OF2) and their synthesized AgNPs (OF2-AgNPs), ascorbic acid was used as a reference compound. The statistical significance level represents *p<0.05 and **p<0.01 (S2 Table).

DPPH assay was utilized to evaluate the antioxidant activity of secondary metabolite (OF2) and synthesized OF2-AgNPs at various concentrations. As result, by increasing concentrations, AgNPs significantly (p<0.05) enhanced the antioxidant potential compared to secondary metabolite extract, as shown in Fig 6. Briefly, in 5mg/mL concentration, the OF2 extract shows 64.8% antioxidant activity while OF2-AgNPs display 99.8% antioxidant activity. In agreement, the GC-MS result of metabolites also revealed the existence of well-known antioxidant compounds like propanedioic acid, phenyl- and 2,3-Butanediol, monooxime [39,40]. Similarly, previous studies also reported that microbial secondary metabolites mediated AgNPs improve biological activities [41,42].

#### Analgesic.

**Fig 7:**
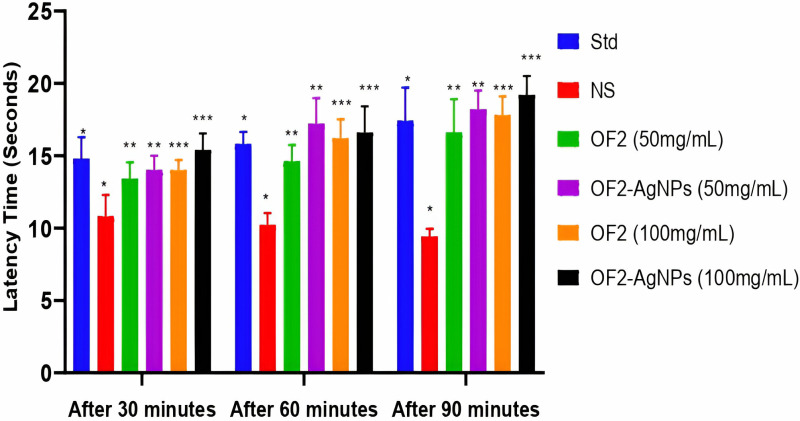
The latency time (seconds) of OF2 secondary metabolites extract and its mediated AgNPs at various concentrations, diclofenac sodium was used as standard (Std) while normal saline (NS) as a control. The statistical significance level represents *p<0.05, **p<0.01, and ***p<0.001 (S4 Table).

The analgesic activity was performed in six groups as described in methods. The latency time in seconds was recorded for all tested animals (S3 Table). The results for tested samples (OF2 extract and OF2-AgNPs) show significant (p<0.05) analgesic effect on tested animals. Fig 7 displays the latency time (seconds) of OF2 extract and OF2 synthesized AgNPs, while in comparison the AgNPs exhibit a higher analgesic effect. Briefly, after 90 minutes at 100 mg/mL concentration, the OF2 extract shows a mean latency time of 17 seconds while OF2-AgNPs show 19 seconds, respectively. In validation of our method, [20,21] also confirmed the analgesic activity of various compounds by our followed method. To our knowledge, it is the first analgesic report of microbial secondary metabolites and their mediated AgNPs. However, the GC-MS results of the metabolites extract confirmed the existence of previously reported analgesic compounds including 2,3-Butanediol, monooxime [43], Tetradecanoic acid [44], 1-Hexanol, 2-ethyl- [45], and 1-Tridecanol [46]. Moreover [47], reported significant analgesic activity of plant-mediated AgNPs.

## Conclusion

In conclusion, *B. amyloliquefaciens* associated with *O. europaea* produced 23 bioactive metabolites. These diverse metabolites have antibacterial, antioxidant, and analgesic activities. Moreover, the AgNPs were successfully synthesized and characterized by using these metabolite extracts, which enhanced these activities. It was suggested from the current study that the microbial secondary metabolite-mediated AgNPs exhibit high pharmaceutical activities. This study will promote the discovery of novel bioactive compounds. Further study prompted; to explore other endophytic microbes associated with *O. europaea*, to explore these metabolites for other biological and pharmaceutical activities. Moreover, the genomic study of the bacterial isolate may explore the biosynthetic pathways for the synthesis of these metabolites.

## Supporting information

S1 Fig *O. europaea* samples. (JPG)

S2 Fig Plant samples (root, Leaves and fruit) on nutrient agar plates. (JPG)

S3 Fig *B. amyloliquefaciens* OF2 *16SrRNA* gene obtained raw sequence .
(JPG)

S1 Table Antibacterial activity of *B. amyloliquefaciens* OF2 secondary metabolites extract and OF2-AgNPs raw data file. (DOCX)

S2 Table DDPH radicals scavenging activity (%) of *B. amyloliquefaciens* derived secondary metabolites (OF2) and their synthesized AgNPs (OF2-AgNPs) raw data file. (DOCX)

S3 Table Pre-treatment reading of 30 selected mice for analgesic activity. (DOCX)

S4 Table *B. amyloliquefaciens* OF2 secondary metabolites extract and its mediated AgNPs at various concentrations analgesic activity raw data file. (DOCX)
