# Supplementary figures and images for: Biological activity of silver nanoparticles synthesized from untapped secondary metabolites of Olea europea endophytic Bacillus amyloliquefaciens
Source: PLoS One. 2025 May 7;20(5):e0321134. doi: 10.1371/journal.pone.0321134 (PMC12057930; doi:10.1371/journal.pone.0321134)

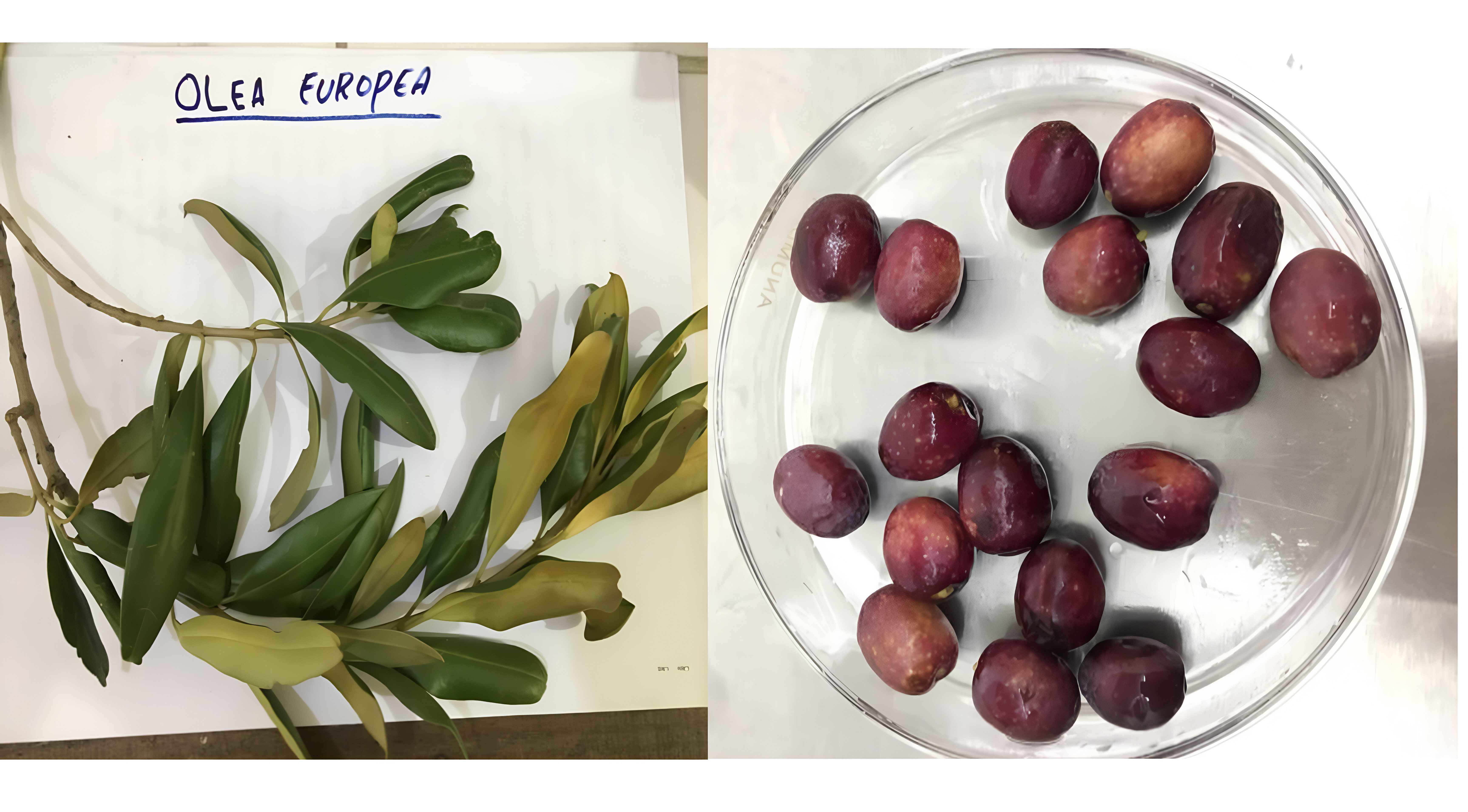

Supplement: S1 Fig — (JPG) [file pone.0321134.s001.jpg]

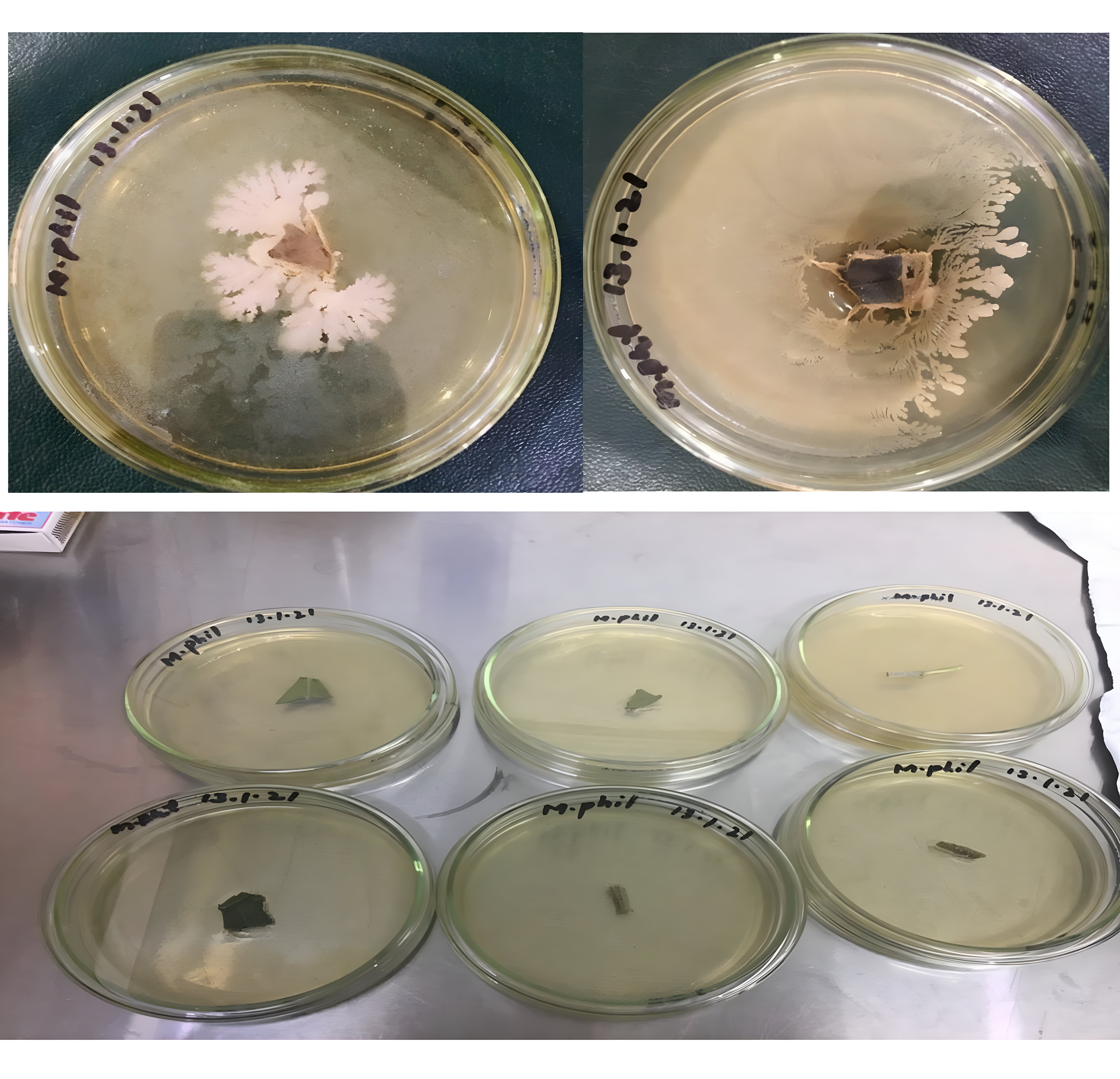

Supplement: S2 Fig — (JPG) [file pone.0321134.s002.jpg]

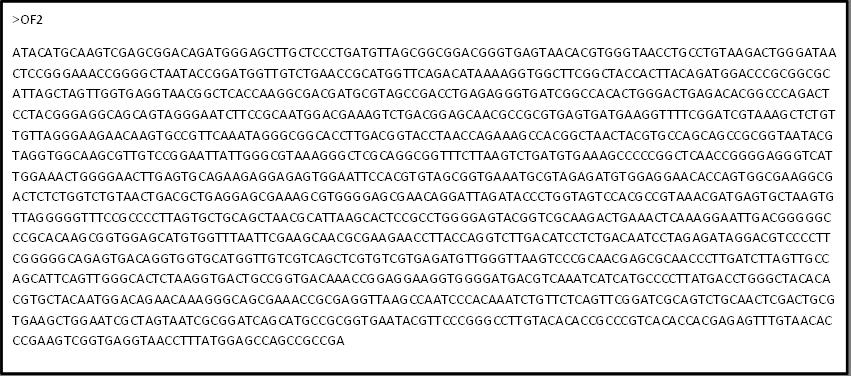

Supplement: S3 Fig — . (JPG) [file pone.0321134.s003.jpg]
