# Supplementary material for: Biological activity of silver nanoparticles synthesized from untapped secondary metabolites of Olea europea endophytic Bacillus amyloliquefaciens
Source: PLoS One. 2025 May 7;20(5):e0321134. doi: 10.1371/journal.pone.0321134 (PMC12057930; doi:10.1371/journal.pone.0321134)
Supplement: S1 Table — (DOCX) [file pone.0321134.s004.docx]

**S1 Table.** Antibacterial activity of *B. amyloliquefaciens* OF2 secondary metabolites extract and OF2-AgNPs raw data file

|  | OF2 extract | OF2 extract | OF2-AgNPs | OF2-AgNPs | Positive control | Positive control | Negative control | Negative control |
| --- | --- | --- | --- | --- | --- | --- | --- | --- |
| *Enterobacteriaceae* | 13 | 13.5 | 14 | 13 | 20 | 19.5 | 0 | 0 |
| *Klebsiella* sp. | 14 | 13 | 16 | 14 | 19 | 19.5 | 0 | 0 |
| *M. morgana* | 19 | 17.5 | 22 | 19.5 | 12 | 11 | 0 | 0 |
| *E. coli* | 19 | 18 | 20 | 17.5 | 24 | 22 | 0 | 0 |
| *P. aeruginosa* | 16 | 15 | 17 | 15.5 | 10 | 9.5 | 0 | 0 |
