# Supplementary material for: Biological activity of silver nanoparticles synthesized from untapped secondary metabolites of Olea europea endophytic Bacillus amyloliquefaciens
Source: PLoS One. 2025 May 7;20(5):e0321134. doi: 10.1371/journal.pone.0321134 (PMC12057930; doi:10.1371/journal.pone.0321134)
Supplement: S2 Table — (DOCX) [file pone.0321134.s005.docx]

**S2 Table.** DDPH radicals scavenging activity (%) of *B. amyloliquefaciens* derived secondary metabolites (OF2) and their synthesized AgNPs (OF2-AgNPs) raw data file

|  | 1 mg/mL | 1 mg/mL | 3 mg/mL | 3 mg/mL | 5 mg/mL | 5 mg/mL |
| --- | --- | --- | --- | --- | --- | --- |
| Ascorbic Acid | 100 | 100 | 100 | 100 | 100 | 100 |
| OF2 | 45 | 45.8 | 54 | 54.8 | 64.8 | 64 |
| OF2-AgNPs | 65 | 65.8 | 72 | 72.8 | 99.8 | 99 |
