# Supplementary material for: Biological activity of silver nanoparticles synthesized from untapped secondary metabolites of Olea europea endophytic Bacillus amyloliquefaciens
Source: PLoS One. 2025 May 7;20(5):e0321134. doi: 10.1371/journal.pone.0321134 (PMC12057930; doi:10.1371/journal.pone.0321134)
Supplement: S3 Table — (DOCX) [file pone.0321134.s006.docx]

**S3 Table.** Pre-treatment reading of 30 selected mice for analgesic activity

| **S. No.** | **Weight (kg)** | **Latency time (Seconds)** | **S. No.** | **Weight (kg)** | **Latency time (Seconds)** |
| --- | --- | --- | --- | --- | --- |
| **1** | 28 | 9 | **16** | 30 | 14 |
| **2** | 30 | 11 | **17** | 25 | 12 |
| **3** | 23 | 10 | **18** | 21 | 10 |
| **4** | 32 | 11 | **19** | 29 | 11 |
| **5** | 30 | 14 | **20** | 36 | 12 |
| **6** | 25 | 12 | **21** | 28 | 9 |
| **7** | 21 | 10 | **22** | 30 | 11 |
| **8** | 29 | 11 | **23** | 23 | 10 |
| **9** | 36 | 12 | **24** | 32 | 11 |
| **10** | 40 | 10 | **25** | 29 | 10 |
| **11** | 31 | 9 | **26** | 27 | 11 |
| **12** | 30 | 14 | **27** | 40 | 10 |
| **13** | 29 | 10 | **28** | 31 | 9 |
| **14** | 27 | 11 | **29** | 30 | 14 |
| **15** | 25 | 12 | **30** | 29 | 10 |
