# Supplementary material for: Biological activity of silver nanoparticles synthesized from untapped secondary metabolites of Olea europea endophytic Bacillus amyloliquefaciens
Source: PLoS One. 2025 May 7;20(5):e0321134. doi: 10.1371/journal.pone.0321134 (PMC12057930; doi:10.1371/journal.pone.0321134)
Supplement: S4 Table — (DOCX) [file pone.0321134.s007.docx]

**S4 Table.** *B. amyloliquefaciens* OF2 secondary metabolites extract and its mediated AgNPs at various concentrations analgesic activity raw data file

|  | Std | Std | Std | NS | NS | NS | OF2 (50mg/mL) | OF2 (50mg/mL) | OF2 (50mg/mL) | OF2-AgNPs (50mg/mL) | OF2-AgNPs (50mg/mL) | OF2-AgNPs (50mg/mL) | OF2 (100mg/mL) | OF2 (100mg/mL) | OF2 (100mg/mL) | OF2-AgNPs (100mg/mL) | OF2-AgNPs (100mg/mL) | OF2-AgNPs (100mg/mL) |
| --- | --- | --- | --- | --- | --- | --- | --- | --- | --- | --- | --- | --- | --- | --- | --- | --- | --- | --- |
| After 30 minutes | 15 | 13 | 17 | 9 | 11 | 13 | 12 | 14 | 15 | 13 | 15 | 14 | 13 | 14 | 15 | 14 | 17 | 16 |
| After 60 minutes | 16 | 16 | 17 | 10 | 11 | 10 | 14 | 15 | 13 | 15 | 16 | 19 | 15 | 15 | 18 | 15 | 16 | 19 |
| After 90 minutes | 18 | 17 | 21 | 9 | 10 | 9 | 16 | 17 | 13 | 17 | 18 | 19 | 17 | 18 | 20 | 18 | 21 | 18 |
